# Potential Mechanisms of Trimetazidine and Coenzyme Q10 Against Antipsychotic‐Induced Myocarditis: A Network Pharmacology, Molecular Docking, and Molecular Dynamics Simulation Study

**DOI:** 10.1002/cns.71009

**Published:** 2026-07-06

**Authors:** Ximing Chen, Dongyin Zhuo, Jiatong Zou, Haitao Song, Kaifang Yao, Hongjun Tian, Chuanjun Zhuo

**Affiliations:** ^1^ Laboratory of Computational Biology and Computational Psychiatry (CBCP‐Lab), Tianjin Anding Hospital Tianjin Mental Health Center, Tianjin Medical University Tianjin China; ^2^ Independent Researcher Technical University Munich Munich Bavaria Germany; ^3^ CBCP‐Lab Tianjin Fourth Center Hospital Tianjin China; ^4^ Department of Psychiatry, Tianjin Fourth Center Hospital Tianjin Medical University Affiliated Tianjin Fourth Center Hospital Tianjin China

**Keywords:** antipsychotic‐induced myocarditis, coenzyme Q10, molecular docking, molecular dynamics simulations, network pharmacology, trimetazidine

## Abstract

**Background:**

Antipsychotic‐induced myocarditis is a rare but potentially fatal adverse event associated with antipsychotic treatment. Trimetazidine (TMZ) and coenzyme Q10 (CoQ10) have shown potential cardioprotective effects. Thus, they may represent adjunctive therapeutic candidates for antipsychotic‐induced myocarditis. However, the underlying molecular mechanisms and therapeutic relevance of this remain unclear. This study aimed to identify the potential pharmacological mechanisms and therapeutic targets of TMZ and CoQ10 in antipsychotic‐induced myocarditis.

**Methods:**

Drug‐ and disease‐associated targets were retrieved or predicted using SwissTargetPrediction, SEA, PharmMapper, Super‐PRED, and GeneCards. Overlapping drug‐disease targets were identified and used to construct a protein–protein interaction network and determine the core targets. Gene Ontology (GO) and Kyoto Encyclopedia of Genes and Genomes (KEGG) enrichment analyses were then performed using DAVID. Finally, molecular docking and molecular dynamics simulations were conducted to evaluate ligand‐target interactions and the stability of the selected complexes.

**Results:**

Twenty‐six overlapping TMZ‐disease targets and 27 overlapping CoQ10‐disease targets were identified. GO and KEGG enrichment analyses revealed that these targets were involved in multiple biological processes, cellular components, molecular functions, and signaling pathways. Thirty‐nine unique drug‐disease intersection targets were obtained after merging the TMZ‐disease and CoQ10‐disease targets and five core targets were identified: STAT3, NFKB1, HIF1A, CASP3, and MMP9. Further GO and KEGG enrichment analyses were conducted for the 39 drug‐disease intersection targets. GO enrichment analysis indicated that the apoptotic process was greatly enriched, whereas KEGG analysis highlighted the PI3K/AKT signaling pathway. Molecular docking suggested that TMZ and CoQ10 could form stable interactions with the core targets, supporting their potential therapeutic relevance. The molecular dynamics simulations further supported the stability of the selected ligand‐target complexes.

**Conclusions:**

Network pharmacology, molecular docking, and molecular dynamics simulations were used to investigate the potential pharmacological mechanisms underlying the effects of TMZ and CoQ10 in antipsychotic‐induced myocarditis. The findings provide theoretical and computational evidence for future experimental studies on TMZ and CoQ10 as potential adjunctive therapeutic candidates for antipsychotic‐induced myocarditis.

## Graphical abstract

In this study, we explored the mechanisms for the therapeutic effects of TMZ and CoQ10 on myocarditis using network pharmacology, molecular docking, and molecular dynamics simulations. Our findings identified core targets of TMZ and CoQ10 treatment of myocarditis; the five core targets included EGFR, MMP9, PPARG, AKT1, and CASP3. GO and KEGG pathway enrichment analyses identified PI3K/AKT signaling as an important pathway involved in antipsychotic‐induced myocarditis. The findings of this study offer novel insights into antipsychotic‐induced myocarditis and provide a theoretical foundation for the development of treatment strategies.

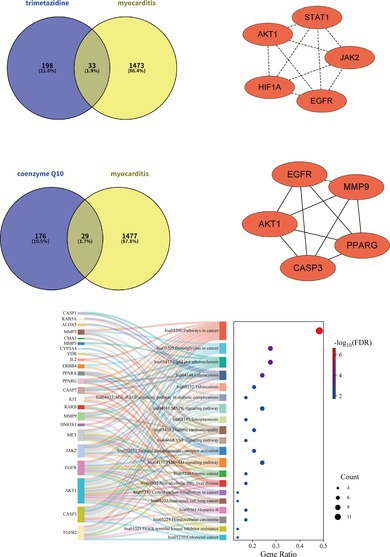

## 1 Introduction

Antipsychotics are evidence‐based first‐line pharmacological treatments for schizophrenia and other primary psychotic disorders [1]. Second‐generation antipsychotic use has increased substantially in recent years, with reported increases often exceeding 50% [2]. However, treatment failure remains a major clinical concern, with one study reporting that 71.1% of patients experience treatment failure [3]. Adverse effects can limit the clinical utility of antipsychotics; therefore, understanding their safety risks is crucial. Myocarditis is a serious and potentially fatal antipsychotic‐associated adverse event [1]. Although several antipsychotic drugs have been associated with myocarditis, clozapine has been most prominently implicated [4, 5]. In an autopsy report of 24 sudden‐death cases, 11 were attributed to myocarditis, including seven clozapine‐treated patients [6]. The incidence of clozapine‐induced myocarditis has been reported to be approximately 5% during the early phase of clozapine treatment [7], and the mortality rate from clozapine‐induced acute myocarditis is about 25% [8].

A systematic review identified several overlapping mechanisms involved in myocarditis development, including increased catecholamine levels, elevated pro‐inflammatory cytokine levels, enhanced reactive oxygen species (ROS) production, reduced antioxidant levels and activity, and mitochondrial damage. Excessive ROS can induce lipid peroxidation, DNA damage, and increase mitochondrial membrane permeability [1, 9, 10]. These processes may exacerbate inflammatory cell infiltration and promote cardiomyocyte apoptosis [11, 12]. Trimetazidine (TMZ) improves cardiomyocyte energy metabolism and possesses antioxidant and cardioprotective properties [13, 14, 15]. Coenzyme Q10 (CoQ10) is a potent antioxidant, inhibiting lipid peroxidation and protecting mitochondria and DNA from oxidative damage. CoQ10 also plays a key role in mitochondrial ATP synthesis [16]. The combination of TMZ and CoQ10 has been reported to improve acute viral myocarditis by alleviating oxidative stress and inflammatory responses [17, 18]. Thus, repurposing TMZ and CoQ10 for drug‐induced myocarditis may be a strategy for patients who require antipsychotic treatment. This study aimed to identify the potential pharmacological mechanisms and candidate therapeutic targets of TMZ and CoQ10 in antipsychotic‐induced myocarditis.

**FIGURE 1:**
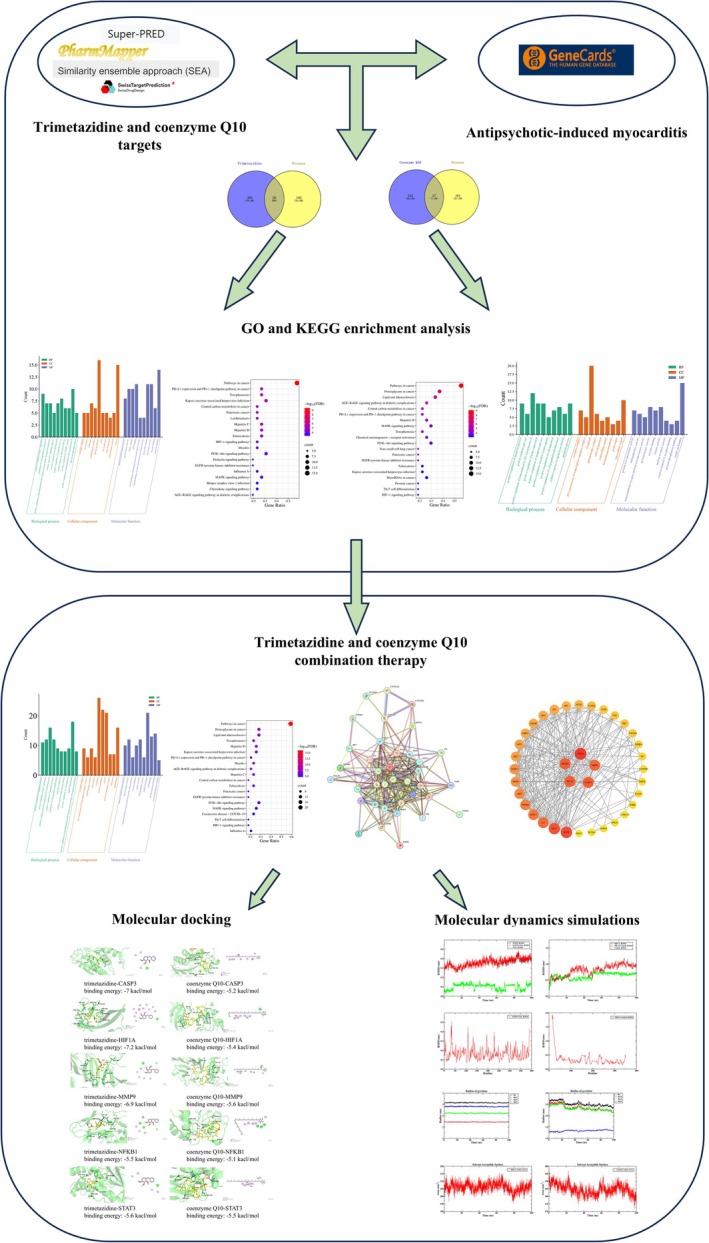
Study workflow.

This study used network pharmacology, molecular docking, and molecular dynamics simulations to systematically investigate the potential mechanisms underlying the effects of TMZ and CoQ10 in treating antipsychotic‐induced myocarditis. Network pharmacology is a useful tool for examining drug‐related molecular mechanisms and pathway‐level regulatory networks [19]. Molecular docking, a computer‐assisted drug design method, is used to predict the binding modes and affinities between ligands and target proteins. Molecular dynamics simulations, which are based on classical mechanics, simulate the atomic‐scale motions of molecular systems by numerically integrating Newton's equations of motion. This approach provides information on molecular structures, dynamic behavior, and intermolecular interactions over a defined timescale. The results of this study provide a theoretical basis for future experimental validation and the therapeutic exploration of antipsychotic‐induced myocarditis. The overall study workflow is shown in Figure 1.

## 2 Methods

### 2.1 Identification of Drug and Disease Targets

The chemical structures and canonical SMILES strings for TMZ and CoQ10 were retrieved from PubChem (https://pubchem.ncbi.nlm.nih.gov/). These strings were subsequently submitted to SwissTargetPrediction [20] (https://swisstargetprediction.ch/) and Super‐PRED [21] (https://prediction.charite.de/), with the organism restricted to *Homo sapiens* and a probability of > 0. TMZ and CoQ10‐related targets were predicted using the SEA [22] (https://sea.bkslab.org/) and PharmMapper databases [23] (https://www.lilab‐ecust.cn/pharmmapper/). The results were restricted to human targets. After the predicted TMZ and CoQ10 targets were merged and deduplicated, the gene symbols were standardized using UniProt [24] (https://www.uniprot.org/). Disease‐associated targets for antipsychotic‐ or clozapine‐induced myocarditis were retrieved from GeneCards [25] (https://www.genecards.org/).

### 2.2 Intersection of Drug and Disease Targets

The predicted targets of each compound were separately intersected with disease‐associated targets to identify the potential therapeutic targets of TMZ and CoQ10 in antipsychotic‐induced myocarditis. The drug and disease target lists were uploaded, and the overlapping targets were identified and visualized using the Venny platform (https://bioinfogp.cnb.csic.es/tools/venny/).

### 2.3 GO and KEGG Enrichment Analyses

Gene Ontology (GO) and Kyoto Encyclopedia of Genes and Genomes (KEGG) pathway enrichment analyses were performed using DAVID [26] (https://davidbioinformatics.nih.gov/). GO terms were categorized into biological processes (BPs), cellular components (CCs), and molecular functions (MFs). *Homo sapiens* was selected as the background organism, and enriched terms or pathways with an FDR of < 0.05 were considered statistically significant.

### 2.4 Protein–Protein Interaction Network Construction and Core Target Selection

Drug‐disease intersection targets were used for subsequent network‐based analysis to elucidate the potential mechanisms underlying the effects of TMZ and CoQ10 in antipsychotic‐induced myocarditis. The drug‐disease intersection targets were obtained by merging the overlapping targets and removing the duplicates. These targets were then imported into STRING [27](https://string‐db.org/) to construct a protein–protein interaction (PPI) network, with the organism set to *H. sapiens* and the minimum required interaction score set to > 0.4. Network topological parameters were analyzed and visualized using Cytoscape 3.9.1 (https://cytoscape.org/). The top five hub targets were identified using the Cytoscape plug‐in CytoHubba [28].

### 2.5 Molecular Docking

The core drug‐disease targets were identified, and their three‐dimensional protein structures were retrieved from the Protein Data Bank [29] (https://www.rcsb.org/). Potential active binding pockets were predicted using POCASA 1.1 (https://g6altair.sci.hokudai.ac.jp/g6/service/pocasa/). Protein structures were prepared by removing water molecules using PyMOL (https://pymol.org/) and adding polar hydrogens using AutoDockTools (https://autodock.scripps.edu/). The TMZ and CoQ10 SDF files were downloaded from PubChem (https://pubchem.ncbi.nlm.nih.gov/), energy‐minimized using ChemDraw 3D, and exported in PDB format. Molecular docking was performed using AutoDock Vina [30]. The docking conformations were visualized using PyMOL and Discovery Studio (https://www.3ds.com/zh‐hans/products/biovia/discovery‐studio).

### 2.6 Molecular Dynamics Simulation

Molecular dynamics simulations were conducted to investigate the conformational dynamics and assess the stability of the ligand‐protein complexes. The most favorable TMZ‐target and CoQ10‐target complexes based on the AutoDock Vina results and defined by the lowest docking binding energies were selected for simulation. A 100‐ns molecular dynamics simulation was performed using GROMACS‐2025.2 [31]. Ligand parameters were generated using the GAFF force field, whereas protein topologies were generated using the CHARMM36 force field [32]. The complexes were solvated in a TIP3P water box, and Na+ ions were added to neutralize the net charge. Energy minimization was performed using the steepest descent method, followed by the conjugate gradient method to obtain a stable minimum‐energy conformation. The system was equilibrated under the NVT and NPT ensembles. The NVT equilibration was performed for 100,000 steps and the NPT equilibration was performed for 100 ps. Production simulations were conducted at 300 K and 1 bar for 50,000,000 steps, with a 2‐fs time step, corresponding to 100 ns.

The root mean square deviation (RMSD) was used to evaluate the global structural deviation of each complex from the reference structure over time. The root mean square fluctuation (RMSF) was calculated to assess residue‐level flexibility during the simulation. The radius of gyration (Rg) was used to assess the overall compactness of each complex during the simulation. Solvent‐accessible surface area (SASA) was calculated to estimate the solvent‐exposed surface area of each complex.

## 3 Results

### 3.1 Predicting Targets for Trimetazidine, Coenzyme Q10, and Antipsychotic Drug‐Induced Myocarditis

**FIGURE 2:**
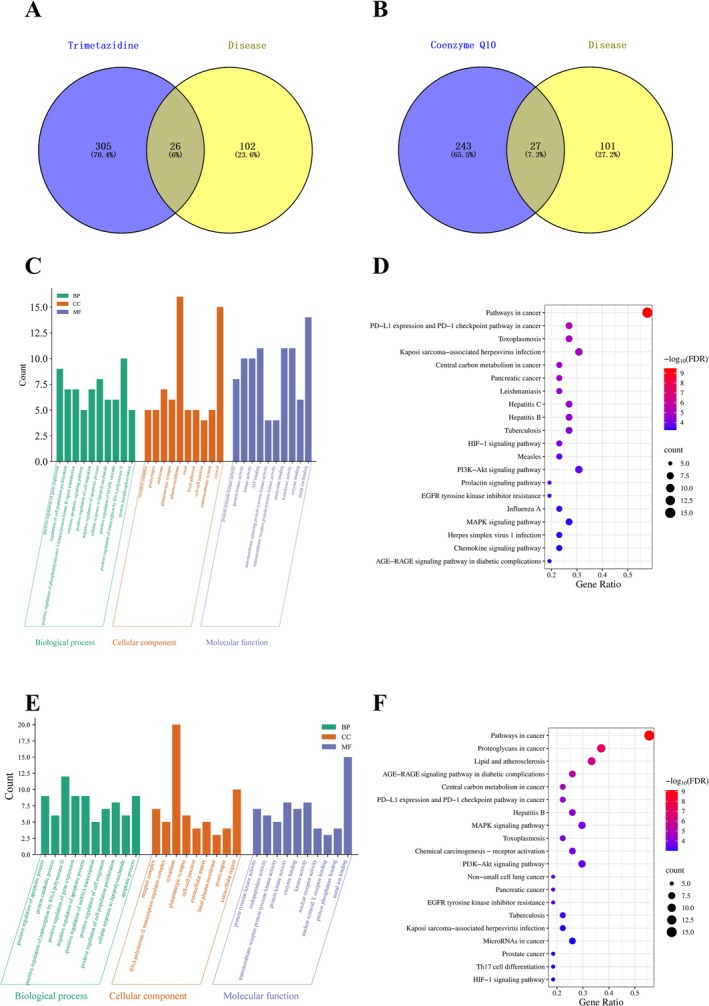
Target prediction and enrichment analysis of TMZ and CoQ10 effects in treating antipsychotic‐induced myocarditis. (A) Venn diagram of TMZ and antipsychotic‐induced myocarditis. (B) Venn diagram of CoQ10 and antipsychotic‐induced myocarditis. (C) Top 10 enriched GO terms associated with TMZ‐disease intersection targets. (D) Top 20 significantly enriched KEGG pathways associated with TMZ‐disease intersection targets. (E) Top 10 enriched GO terms associated with CoQ10‐disease intersection targets. (F) Top 20 significantly enriched KEGG pathways associated with CoQ10‐disease intersection targets.

A total of 331 TMZ‐related targets, 270 CoQ10‐related targets, and 128 targets associated with antipsychotic‐ or clozapine‐induced myocarditis were identified. The overlap between drug‐related and disease‐related targets was analyzed using Venny 2.1.0, in which 26 overlapping TMZ‐disease targets (Figure 2A) and 27 overlapping CoQ10‐disease targets (Figure 2B) were identified.

### 3.2 GO and KEGG Pathway Analyses

DAVID‐based enrichment analysis of the overlapping TMZ‐disease targets identified 171 BP terms, 30 CC terms, 28 MF terms, and 84 KEGG pathways. Based on FDR ranking, the most significantly enriched BP, CC, and MF terms were the positive regulation of gene expression, receptor complex, and protein tyrosine kinase activity, respectively (Figure 2C). After disease‐specific pathways were excluded, the major enriched signaling pathways included the HIF‐1 signaling pathway and the PI3K‐Akt signaling pathway (Figure 2D). A total of 200 BP terms, 22 CC terms, 45 MF terms, and 91 KEGG pathways were identified for the overlapping CoQ10‐disease targets. The most significantly enriched BP, CC, and MF terms were the apoptotic process, receptor complex, and protein tyrosine kinase activity, respectively (Figure 2E). After disease‐specific pathways were excluded, the major enriched pathways included lipid and atherosclerosis and the PI3K‐Akt signaling pathway (Figure 2F).

### 3.3 Mechanistic Analysis of TMZ and CoQ10 in Antipsychotic‐Induced Myocarditis

#### 3.3.1 Construction of the PPI Network and Identification of Core Targets

**FIGURE 3:**
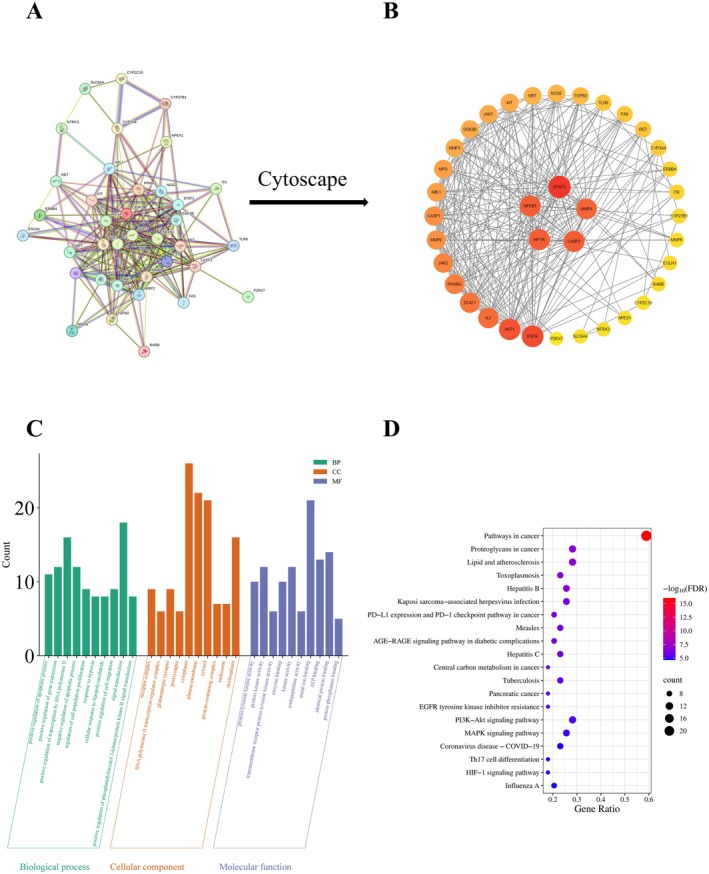
Potential mechanisms of TMZ and CoQ10 in antipsychotic‐induced myocarditis. (A) Protein–protein interaction (PPI) network of drug‐disease intersection targets. (B) Core targets in the drug‐disease network. Brighter node colors indicate greater topological importance, and nodes in the inner circle represent the core targets. (C) Top 10 enriched GO terms associated with the drug‐disease intersection targets. (D) Top 20 significant KEGG signaling pathways associated with the drug‐disease intersection targets.

The TMZ‐disease and CoQ10‐disease targets were merged, yielding 39 unique drug‐disease intersection targets. The PPI network of these 39 drug‐disease intersection targets contained 39 nodes and 250 edges (Figure 3A). After disconnected nodes were removed, the resulting network was visualized using Cytoscape 3.9.1 (Figure 3B). The identified core targets associated with the drug‐disease network were STAT3, NFKB1, HIF1A, CASP3, and MMP9 (Figure 3B).

#### 3.3.2 GO and KEGG Pathway Analyses

GO analysis revealed the enrichment of 260 BP terms, 36 CC terms, and 56 MF terms. The top 10 significantly enriched GO terms with an FDR of < 0.05 are shown in Figure 3C. The most significantly enriched BP terms were associated with the apoptotic process. The most significantly enriched CC terms included receptor complex. The most significantly enriched MF terms were associated with protein tyrosine kinase activity (Figure 3C). The top 20 enriched pathways among the 97 identified KEGG pathways are shown in Figure 3D. After disease‐specific pathways were excluded, the most enriched pathways were lipid and atherosclerosis and the PI3K‐Akt signaling pathway (Figure 3D).

### 3.4 Molecular Docking

**FIGURE 4:**
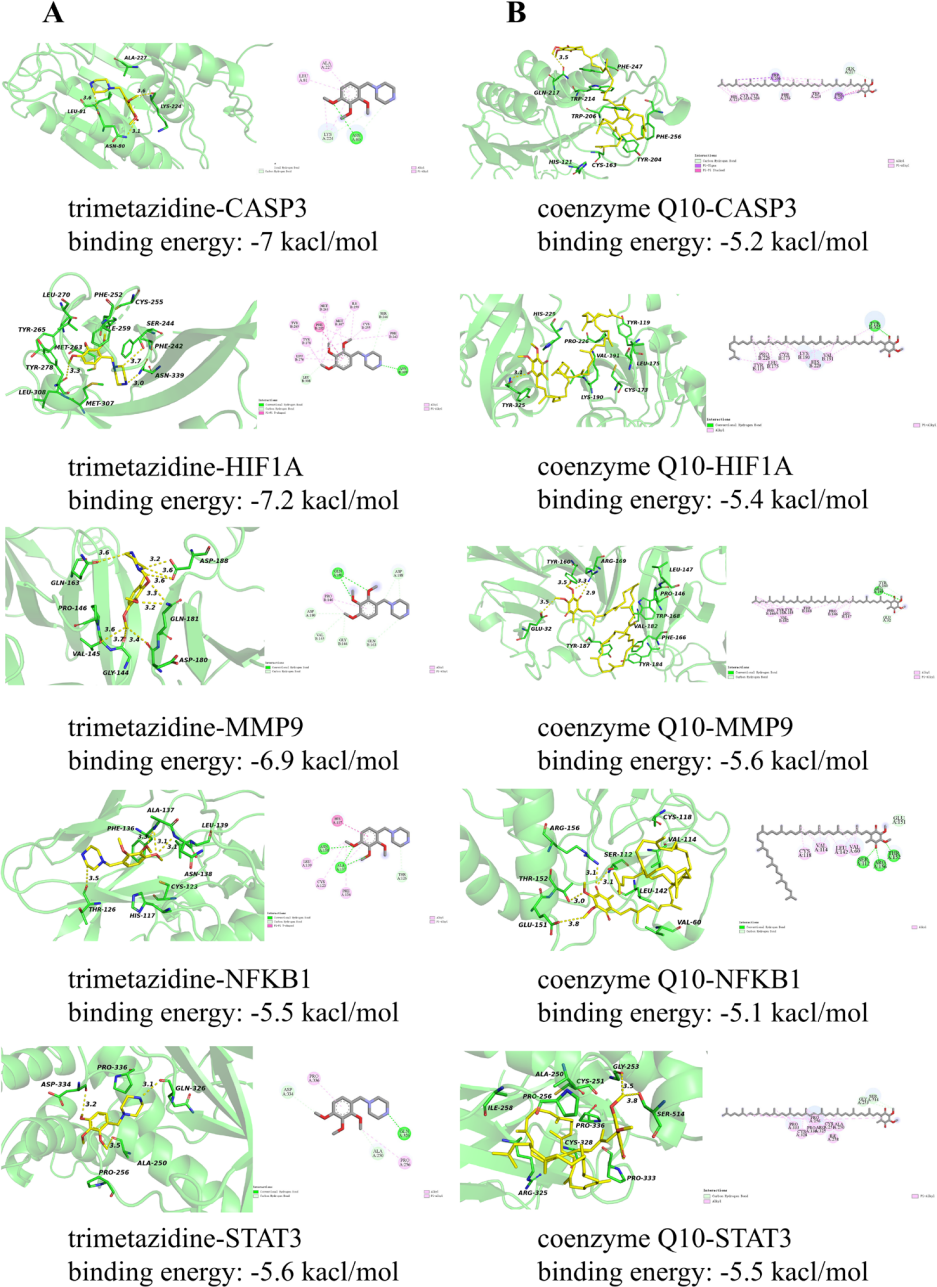
Molecular docking results of TMZ and CoQ10 with the identified hub targets. (A) Binding conformations and docking scores of TMZ with the core targets. (B) Binding conformations and docking scores of CoQ10 with the core targets.

The five core targets shared by TMZ, CoQ10, and antipsychotic‐induced myocarditis were selected for molecular docking: STAT3 (PDB ID: 6NJS), NFKB1 (PDB ID: 8TQD), HIF1A (PDB ID: 9A9Z), MMP9 (PDB ID: 1ITV), and CASP3 (PDB ID: 3PCX) (Figure 4). Complexes with binding energies between −5 and −7 kcal/mol were considered to exhibit stable docking, whereas those with binding energies of ≤ −7 kcal/mol were considered to exhibit strong docking [33]. The docking results of all ligand‐protein complexes exhibited acceptable binding affinities according to these criteria.

### 3.5 Molecular Dynamics Simulations of Potential Targets

**FIGURE 5:**
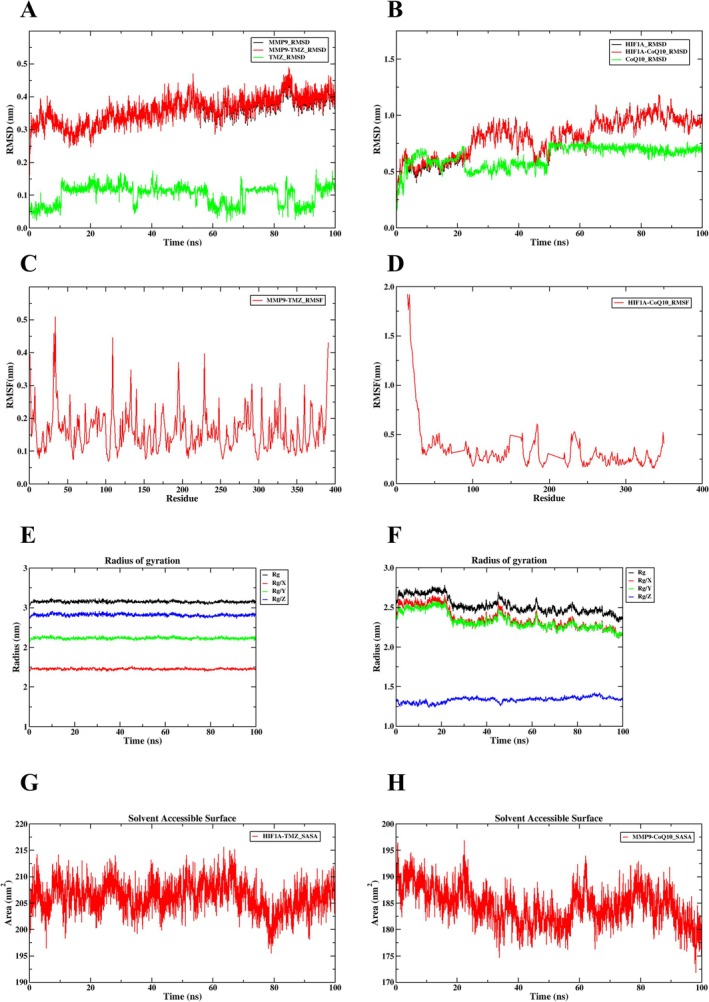
Molecular dynamics simulation analysis of the TMZ‐MMP9 and CoQ10‐HIF1A complexes. (A) Root mean square deviation (RMSD) of TMZ‐MMP9. (B) RMSD of CoQ10‐HIF1A. (C) Root mean square fluctuation (RMSF) of TMZ‐MMP9. (D) RMSF of CoQ10‐HIF1A. (E) Radius of gyration (Rg) of TMZ‐MMP9. (F) Rg of CoQ10‐HIF1A. (G) Solvent‐accessible surface area (SASA) of TMZ‐MMP9. (H) SASA of CoQ10‐HIF1A.

Molecular dynamics simulations were performed to further evaluate the stability of the docking complexes, including MMP9‐TMZ (PDB ID: 1ITV) and HIF1A‐CoQ10 (PDB ID: 9A9Z). The MMP9‐TMZ system displayed relatively stable conformational dynamics during the 100‐ns simulation. The RMSD curves of the MMP9 backbone and the MMP9‐TMZ complex largely overlapped and remained mainly within 0.35–0.45 nm, indicating that TMZ binding did not substantially perturb the overall backbone conformation of MMP9 (Figure 5A). In contrast, the HIF1A‐CoQ10 system exhibited RMSD fluctuations. The RMSD of the HIF1A‐CoQ10 complex increased during the early and middle phases of the simulation and reached a relatively stable plateau of approximately 1.0 nm after 65 ns (Figure 5B). The results suggest that both complexes reached apparent dynamic equilibrium, although the HIF1A‐CoQ10 complex exhibited greater structural flexibility than the MMP9‐TMZ complex. The RMSF analysis indicated that the major structural regions in both complexes remained relatively stable during the simulation. The MMP9‐TMZ complex displayed lower overall flexibility, with most residues showing RMSF values less than 0.3 nm and only a few localized peaks approaching 0.5 nm (Figure 5C). The HIF1A‐CoQ10 complex showed marked flexibility in the N‐terminal region, with RMSF values reaching approximately 1.9 nm, whereas most other residues fluctuated below 0.6 nm (Figure 5D). These findings suggest that both complexes maintained stable core conformations, with flexibility mainly confined to terminal segments or local loop regions. Rg analysis indicated that the MMP9‐TMZ complex maintained a stable compactness profile during the 100‐ns simulation, with only minor fluctuations in the overall Rg value (Figure 5E). In contrast, the Rg of the HIF1A‐CoQ10 complex notably decreased during the early phase of the simulation, followed by moderate fluctuations and subsequent stabilization, suggesting conformational compaction before equilibration (Figure 5F). The results indicate that both complexes preserved their overall structural integrity, although the HIF1A‐CoQ10 complex displayed greater compactness‐related rearrangement than the MMP9‐TMZ complex. Additionally, the SASA analysis indicated relatively stable solvent exposure in both systems. The MMP9‐TMZ complex remained mainly within 200–212 nm^2^, accompanied by moderate fluctuations but without persistent expansion (Figure 5G). The HIF1A‐CoQ10 complex maintained a relatively stable solvent‐accessible surface area, with values mainly fluctuating between 175 and 195 nm^2^ and showing a slight downward trend during the 100‐ns simulation (Figure 5H). The findings suggest that both complexes retained relatively stable surface exposure during the simulation, further supporting the stability of the predicted docking conformations.

## 4 Discussion

Antipsychotic‐induced myocarditis is a rare but potentially fatal adverse event associated with antipsychotic treatment, particularly during the first few months after treatment initiation. Effective management includes clinical monitoring for myocarditis and decreasing or discontinuing antipsychotic medications, and adjunctive pharmacological interventions may be necessary to control myocardial inflammation and injury. The combination of TMZ and CoQ10 has been investigated as a potential therapeutic strategy for acute viral myocarditis. Therefore, we explored the potential molecular mechanisms underlying the therapeutic effects of TMZ and CoQ10 in antipsychotic‐induced myocarditis.

Thirty‐nine unique drug‐disease targets potentially involved in antipsychotic‐induced myocarditis were identified by merging 26 TMZ‐disease intersection targets and 27 CoQ10‐disease intersection targets. PPI network construction and topological analysis identified five core targets in the drug‐disease network, namely STAT3, NFKB1, HIF1A, CASP3, and MMP9. Molecular docking analysis further indicated that the candidate ligands could stably bind to these hub targets. Molecular dynamics simulations were then conducted to assess the dynamic stability of selected ligand‐protein complexes during a 100‐ns simulation. Previous studies suggested that STAT3 plays a cardioprotective role and may attenuate myocarditis [34]. STAT3 has also been regarded as a potential therapeutic target for viral myocarditis [35]. Activation of the JAK2/STAT3 pathway has been associated with reduced arrhythmias, cardiac inflammation, and myocardial injury [36, 37]. TMZ was reported to alleviate oxidative stress and inflammation in rats with ovarian ischemia–reperfusion injury via the JAK2/STAT3 signaling pathway [38]. CoQ10 was found to significantly decrease the levels of pro‐inflammatory cytokines and STAT3 expression [39]. The suppression of NF‐κB signaling has been reported to improve acute viral myocarditis [40] and autoimmune myocarditis [41]. Furthermore, TMZ has shown therapeutic potential in rheumatoid arthritis [42], neuroinflammation [43] and ulcerative colitis [44], partly by downregulating NFKB1. Our findings suggest that NFKB1 may contribute to the potential therapeutic effects of TMZ in antipsychotic‐induced myocarditis. In addition, a previous study suggested that CoQ10 could induce the downregulation of HIF1A and NFKB1 [45]. HIF1A expression is elevated in patients with acute autoimmune myocarditis [46], and HIF1A can activate pro‐inflammatory macrophages, thereby promoting the progression of autoimmune myocarditis [47]. TMZ has been reported to activate the HIF1A/HO‐1 signaling pathway, leading to improved hemodynamics and decreased fibrosis and inflammation [48]. CASP3 plays a central role in cell apoptosis, necrosis, and inflammation [49]. Increased CASP3 expression has been observed in myocardial infarction and myocarditis [50, 51]. A recent myocarditis study reported that topotecan could reduce CASP3 expression by inhibiting HIF1A [52]. MMP9 plays a crucial role in acute autoimmune myocarditis, and MMP9 inhibition may attenuate myocardial inflammation [53]. TMZ has been shown to decrease MMP9 expression and attenuate myocardial infarction‐induced oxidative stress [54]. CoQ10 decreases inflammatory markers, including tumor necrosis factor‐α, interleukin (IL)‐6, and MMP9 [55], and improves mitochondrial function [56]. The cardiac expression of vimentin, connexin‐43, and CASP3 was reported to increase in rat models of clozapine‐induced myocarditis [57]. CoQ10 significantly inhibits CASP3 expression, thereby mitigating the adverse effects of drug‐induced cardiotoxicity [58, 59]. In this study, network pharmacology identified multiple candidate therapeutic targets potentially involved in antipsychotic‐induced myocarditis. The findings suggest that CoQ10 and TMZ may modulate key inflammatory, apoptotic, and metabolic signaling pathways and thereby reduce myocarditis‐related injury.

GO enrichment analysis suggested that the apoptotic process, receptor complex, and protein tyrosine kinase activity may play important roles in the effects of TMZ and CoQ10 in treating antipsychotic‐induced myocarditis. In myocarditis, inflammatory stimuli can activate apoptotic processes, exerting deleterious effects on cardiac tissue [60]. TMZ and CoQ10 have been reported to possess antioxidant, anti‐apoptotic, and anti‐inflammatory properties [61, 62]. Our findings suggest that TMZ and CoQ10 may exert therapeutic effects on antipsychotic‐induced myocarditis partly through anti‐apoptotic mechanisms. In addition, KEGG pathway analysis indicated that TMZ and CoQ10 may be involved in myocarditis‐related regulation via the PI3K/AKT signaling pathway. Therefore, the PI3K/AKT signaling pathway may represent a key mechanistic axis in the predicted therapeutic effects of TMZ and CoQ10. Previous studies also support the involvement of the PI3K/AKT signaling pathway in myocarditis. Macrostemonoside T has been shown to increase the expression of phosphorylated (p)‐PI3K, p‐AKT, and p‐mTOR, thereby protecting myocardial cells [63]. Inhibiting PI3K significantly reduces PI3K/AKT signaling in vivo, leading to the upregulation of the CD73‐adenosine axis and apoptosis, and thereby aggravating myocarditis [64]. Furthermore, activation of the PI3K/AKT signaling pathway has been reported to reduce cardiac inflammation and mitochondrial injury [65]. Numerous studies have highlighted the critical role of the PI3K/AKT/mTOR signaling pathway in regulating apoptosis and autophagy [66, 67]. Additionally, activating the PI3K/AKT/mTOR pathway in neuronal cells has been shown to reduce oxidative stress and alleviate neuroinflammation [68]. Kamranian et al. demonstrated that TMZ regulates the PI3K/AKT/mTOR signaling pathway to inhibit key biological processes, including apoptosis, autophagy, inflammation, mitochondrial dysfunction, and oxidative stress [69]. CoQ10 has been reported to inhibit oxidative stress and modulate the PI3K/AKT pathway, thereby providing neuroprotective effects [70]. In animal studies, insulin protected cardiac mitochondrial morphology via the PI3K/AKT signaling pathway [71]. Wen et al. [72] demonstrated that CoQ10 improved cardiac myocyte apoptosis by decreasing the expression of AKT and PI3K. Taken together, TMZ and CoQ10 may exert complementary therapeutic effects through PI3K/AKT‐related signaling in antipsychotic‐induced myocarditis, providing a theoretical basis for their combined use.

In conclusion, network pharmacology, molecular docking, and molecular dynamics simulations were employed to explore the potential molecular mechanisms underlying the effects of TMZ and CoQ10 in treating antipsychotic‐induced myocarditis. Several candidate core targets associated with TMZ and CoQ10 treatment of myocarditis were identified. GO and KEGG pathway enrichment analyses identified the PI3K/AKT signaling as a potentially important pathway involved in the predicted therapeutic effects of TMZ and CoQ10 in antipsychotic‐induced myocarditis. The findings provide mechanistic insight into antipsychotic‐induced myocarditis and a theoretical basis for developing adjunctive therapeutic strategies. Network pharmacology provides a useful approach for elucidating complex molecular links between drugs and diseases, while molecular docking and molecular dynamics simulations can further assess ligand‐target interactions and complex stability. However, the study findings require further validation using in vitro and in vivo models of antipsychotic‐induced myocarditis.

## Author Contributions

Ximing Chen: data curation, formal analysis, investigation, and writing – original draft. Chuanjun Zhuo and Hongjun Tian: conceptualization, formal analysis, funding acquisition, methodology, supervision, writing – original draft, and writing – review and editing. Haitao Song: formal analysis, investigation, and writing – review and editing. Jiatong Zou: formal analysis, investigation, and writing – review and editing. Kaifang Yao: formal analysis, investigation, and writing – review and editing.

## Funding

This work was sponsored by an award from the National Natural Science Foundation of China : 82171503 and 81871052 to Chuanjun Zhuo, Tianjin Anding Hospital Talent Fundation to Chuanjun Zhuo (300,000 YUAN RMB) and Tianjin Health Research Project: TJWJ2025ZK009 to Hongjun Tian.

## Ethics Statement

This study did not require ethical approval because the analysis only included data uploaded from public database searches.

## Conflicts of Interest

The authors declare no conflicts of interest.

## Data Availability

The data that support the findings of this study are available from the corresponding author upon reasonable request.

## References

[1] N. Vaziri , D. Marques , S. C. Greenway , and C. A. Bousman , “The Cellular Mechanism of Antipsychotic‐Induced Myocarditis: A Systematic Review,” Schizophrenia Research 261 (2023): 206–215. 37797362 10.1016/j.schres.2023.09.039

[2] J. Ying , Q. H. Chew , Y. Wang , and K. Sim , “Global Neuropsychopharmacological Prescription Trends in Adults With Schizophrenia, Clinical Correlates and Implications for Practice: A Scoping Review,” Brain Sciences 14, no. 1 (2023): 6. 38275511 10.3390/brainsci14010006 PMC10813099

[3] A. Hamina , H. Taipale , J. Lieslehto , et al., “Comparative Effectiveness of Antipsychotics in Patients With Schizophrenia Spectrum Disorder,” JAMA Network Open 7, no. 10 (2024): e2438358. 39382894 10.1001/jamanetworkopen.2024.38358 PMC11465102

[4] X. Q. Li , X. R. Tang , and L. L. Li , “Antipsychotics Cardiotoxicity: What's Known and What's Next,” World Journal of Psychiatry 11, no. 10 (2021): 736–753. 34733639 10.5498/wjp.v11.i10.736 PMC8546771

[5] M. Qubad , G. Dupont , M. Hahn , et al., “When, Why and How to re‐Challenge Clozapine in Schizophrenia Following Myocarditis,” CNS Drugs 38, no. 9 (2024): 671–696. 38951464 10.1007/s40263-024-01100-4 PMC11316720

[6] L. Li , X. Ye , Z. Zhao , P. Gao , and Y. Jiang , “Overlooked Fatal Infectious Diseases After Long‐Term Antipsychotic Use in Patients With Psychiatric Illness,” Schizophrenia Research 195 (2018): 258–259. 29128324 10.1016/j.schres.2017.09.033

[7] S. Sandarsh , R. J. Bishnoi , R. B. Shashank , B. J. Miller , O. Freudenreich , and J. P. McEvoy , “Monitoring for Myocarditis During Treatment Initiation With Clozapine,” Acta Psychiatrica Scandinavica 144, no. 2 (2021): 194–200. 33997951 10.1111/acps.13328

[8] M. Vickers , V. Ramineni , E. Malacova , et al., “Risk Factors for Clozapine‐Induced Myocarditis and Cardiomyopathy: A Systematic Review and Meta‐Analysis,” Acta Psychiatrica Scandinavica 145, no. 5 (2022): 442–455. 35067911 10.1111/acps.13398

[9] P. R. Angelova , A. J. C. Kerbert , A. Habtesion , A. Hall , A. Y. Abramov , and R. Jalan , “Hyperammonaemia Induces Mitochondrial Dysfunction and Neuronal Cell Death,” JHEP Reports 4, no. 8 (2022): 100510. 35845295 10.1016/j.jhepr.2022.100510 PMC9278080

[10] Z. Wu , H. Wang , S. Fang , and C. Xu , “Roles of Endoplasmic Reticulum Stress and Autophagy on H_2_O_2_‐Induced Oxidative Stress Injury in HepG2 Cells,” Molecular Medicine Reports 18, no. 5 (2018): 4163–4174. 30221706 10.3892/mmr.2018.9443 PMC6172379

[11] C. Holze , C. Michaudel , C. Mackowiak , et al., “Oxeiptosis, a ROS‐Induced Caspase‐Independent Apoptosis‐Like Cell‐Death Pathway,” Nature Immunology 19, no. 2 (2018): 130–140. 29255269 10.1038/s41590-017-0013-y PMC5786482

[12] X. Fan , T. Dong , K. Yan , X. Ci , and L. Peng , “PM2.5 Increases Susceptibility to Acute Exacerbation of COPD via NOX4/Nrf2 Redox Imbalance‐Mediated Mitophagy,” Redox Biology 59 (2023): 102587. 36608590 10.1016/j.redox.2022.102587 PMC9813701

[13] M. Marzilli , D. Vinereanu , G. Lopaschuk , et al., “Trimetazidine in Cardiovascular Medicine,” International Journal of Cardiology 293 (2019): 39–44. 31178223 10.1016/j.ijcard.2019.05.063

[14] F. Kayan and H. B. Savas , “Effects of Trimetazidine on Oxidant‐Antioxidant Balance and Angiogenesis; an In Vivo Experimental Study,” BMC Cardiovascular Disorders 25, no. 1 (2025): 275. 40211152 10.1186/s12872-025-04701-z PMC11983985

[15] Y. Li , Y. Li , Z. Zhang , et al., “Efficacy and Safety of Yangxinshi Versus Trimetazidine on Exercise Tolerance in Patients With Coronary Heart Disease After Percutaneous Coronary Intervention: Multicenter, Double‐Blind Clinical Trial,” Phytomedicine 135 (2024): 156198. 39566404 10.1016/j.phymed.2024.156198

[16] M. Arenas‐Jal , J. M. Suñé‐Negre , and E. García‐Montoya , “Coenzyme Q10 Supplementation: Efficacy, Safety, and Formulation Challenges,” Comprehensive Reviews in Food Science and Food Safety 19, no. 2 (2020): 574–594. 33325173 10.1111/1541-4337.12539

[17] Y. J. Yin , S. L. Zeng , Y. W. Li , Z. Wu , D. J. Huang , and H. Z. Tang , “The Effect of Coenzyme Q10 Plus Trimetazidine on Acute Viral Myocarditis Treatment,” American Journal of Translational Research 13, no. 12 (2021): 13854–13861. 35035725 PMC8748115

[18] L. Shao , A. Ma , G. Figtree , and P. Zhang , “Combination Therapy With Coenzyme Q10 and Trimetazidine in Patients With Acute Viral Myocarditis,” Journal of Cardiovascular Pharmacology 68, no. 2 (2016): 150–154. 27046339 10.1097/FJC.0000000000000396

[19] C. Nogales , Z. M. Mamdouh , M. List , C. Kiel , A. I. Casas , and H. Schmidt , “Network Pharmacology: Curing Causal Mechanisms Instead of Treating Symptoms,” Trends in Pharmacological Sciences 43, no. 2 (2022): 136–150. 34895945 10.1016/j.tips.2021.11.004

[20] A. Daina , O. Michielin , and V. Zoete , “SwissTargetPrediction: Updated Data and New Features for Efficient Prediction of Protein Targets of Small Molecules,” Nucleic Acids Research 47, no. W1 (2019): W357–w364. 31106366 10.1093/nar/gkz382 PMC6602486

[21] K. Gallo , A. Goede , R. Preissner , and B. O. Gohlke , “SuperPred 3.0: Drug Classification and Target Prediction—A Machine Learning Approach,” Nucleic Acids Research 50, no. W1 (2022): W726–w731. 35524552 10.1093/nar/gkac297 PMC9252837

[22] M. J. Keiser , B. L. Roth , B. N. Armbruster , P. Ernsberger , J. J. Irwin , and B. K. Shoichet , “Relating Protein Pharmacology by Ligand Chemistry,” Nature Biotechnology 25, no. 2 (2007): 197–206. 10.1038/nbt1284 17287757

[23] X. Wang , Y. Shen , S. Wang , et al., “PharmMapper 2017 Update: A Web Server for Potential Drug Target Identification With a Comprehensive Target Pharmacophore Database,” Nucleic Acids Research 45, no. W1 (2017): W356–W360. 28472422 10.1093/nar/gkx374 PMC5793840

[24] S. Ahmad , L. Jose da Costa Gonzales , E. H. Bowler‐Barnett , et al., “The UniProt Website API: Facilitating Programmatic Access to Protein Knowledge,” Nucleic Acids Research 53, no. W1 (2025): W547–W553. 40331428 10.1093/nar/gkaf394 PMC12230682

[25] G. Stelzer , N. Rosen , I. Plaschkes , et al., “The GeneCards Suite: From Gene Data Mining to Disease Genome Sequence Analyses,” Current Protocols in Bioinformatics 54 (2016): 1.30.1–1.30.33. 10.1002/cpbi.5 27322403

[26] B. T. Sherman , M. Hao , J. Qiu , et al., “DAVID: A Web Server for Functional Enrichment Analysis and Functional Annotation of Gene Lists (2021 Update),” Nucleic Acids Research 50, no. W1 (2022): W216–W221. 35325185 10.1093/nar/gkac194 PMC9252805

[27] D. Szklarczyk , R. Kirsch , M. Koutrouli , et al., “The STRING Database in 2023: Protein‐Protein Association Networks and Functional Enrichment Analyses for Any Sequenced Genome of Interest,” Nucleic Acids Research 51, no. D1 (2023): D638–D646. 36370105 10.1093/nar/gkac1000 PMC9825434

[28] C. H. Chin , S. H. Chen , H. H. Wu , C. W. Ho , M. T. Ko , and C. Y. Lin , “cytoHubba: Identifying Hub Objects and Sub‐Networks From Complex Interactome,” BMC Systems Biology 8, no. Suppl 4 (2014): S11. 25521941 10.1186/1752-0509-8-S4-S11 PMC4290687

[29] S. K. Burley , R. Bhatt , C. Bhikadiya , et al., “Updated Resources for Exploring Experimentally‐Determined PDB Structures and Computed Structure Models at the RCSB Protein Data Bank,” Nucleic Acids Research 53, no. D1 (2025): D564–D574. 39607707 10.1093/nar/gkae1091 PMC11701563

[30] O. Trott and A. J. Olson , “AutoDock Vina: Improving the Speed and Accuracy of Docking With a New Scoring Function, Efficient Optimization, and Multithreading,” Journal of Computational Chemistry 31, no. 2 (2010): 455–461. 19499576 10.1002/jcc.21334 PMC3041641

[31] M. J. Abraham , T. Murtola , R. Schulz , et al., “GROMACS: High Performance Molecular Simulations Through Multi‐Level Parallelism From Laptops to Supercomputers,” SoftwareX 1‐2 (2015): 19–25.

[32] K. Vanommeslaeghe , E. Hatcher , C. Acharya , et al., “CHARMM General Force Field: A Force Field for Drug‐Like Molecules Compatible With the CHARMM All‐Atom Additive Biological Force Fields,” Journal of Computational Chemistry 31, no. 4 (2010): 671–690. 19575467 10.1002/jcc.21367 PMC2888302

[33] Z. Du , Y. Zeng , Z. Zhao , et al., “Integrative Approaches to Uncover the Therapeutic Action of Huaiqihuang in Myocarditis: Network Pharmacology, Molecular Docking, and Molecular Dynamics,” Current Pharmaceutical Design 32 (2026): e101651. 10.2174/0113816128393399251021101651 41568488

[34] M. Kurdi , C. Zgheib , and G. W. Booz , “Recent Developments on the Crosstalk Between STAT3 and Inflammation in Heart Function and Disease,” Frontiers in Immunology 9 (2018): 3029. 30619368 10.3389/fimmu.2018.03029 PMC6305745

[35] T. Liang , Z. Zhang , Z. Bai , L. Xu , and W. Xu , “STAT3 Increases CVB3 Replication and Acute Pancreatitis and Myocarditis Pathology via Impeding Nuclear Translocation of STAT1 and Interferon‐Stimulated Gene Expression,” International Journal of Molecular Sciences 25, no. 16 (2024): 9007. 39201692 10.3390/ijms25169007 PMC11354559

[36] H. Park , H. Park , D. Mun , et al., “Sympathetic Nerve Blocks Promote Anti‐Inflammatory Response by Activating the JAK2‐STAT3‐Mediated Signaling Cascade in Rat Myocarditis Models: A Novel Mechanism With Clinical Implications,” Heart Rhythm 15, no. 5 (2018): 770–779. 28963014 10.1016/j.hrthm.2017.09.039

[37] T. Xia , T. Wu , T. Wu , Y. Ren , Z. Wang , and R. Wu , “Tanshinone Attenuates Myocardial Injury via Activating JAK2/STAT1 Pathway in a Murine Model of Viral Myocarditis,” Zhonghua Xin Xue Guan Bing Za Zhi 43, no. 2 (2015): 167–172. 25907491

[38] T. N. Yuksel , Z. Halici , E. Cadirci , E. Toktay , B. Ozdemir , and A. Bozkurt , “Effect of Trimetazidine Against Ovarian Ischemia/Reperfusion Injury in Rat Model: A New Pathway: JAK2/STAT3,” Iranian Journal of Basic Medical Sciences 26, no. 11 (2023): 1370–1379. 37886007 10.22038/IJBMS.2023.72544.15776 PMC10598820

[39] J. Jhun , J. Moon , J. Ryu , et al., “Liposome/Gold Hybrid Nanoparticle Encoded With CoQ10 (LGNP‐CoQ10) Suppressed Rheumatoid Arthritis via STAT3/Th17 Targeting,” PLoS One 15, no. 11 (2020): e0241080. 33156836 10.1371/journal.pone.0241080 PMC7647073

[40] Y. Xue , T. Song , J. Ke , et al., “MG53 Protects Against Coxsackievirus B3‐Induced Acute Viral Myocarditis in Mice by Inhibiting NLRP3 Inflammasome‐Mediated Pyroptosis via the NF‐κB Signaling Pathway,” Biochemical Pharmacology 223 (2024): 116173. 38552849 10.1016/j.bcp.2024.116173

[41] T. Gan , W. Liu , Y. Wang , et al., “LncRNA MAAMT Facilitates Macrophage Recruitment and Proinflammatory Activation and Exacerbates Autoimmune Myocarditis Through the SRSF1/NF‐κB Axis,” International Journal of Biological Macromolecules 278, no. Pt 1 (2024): 134193. 39069042 10.1016/j.ijbiomac.2024.134193

[42] E. Omran , A. R. Alzahrani , S. F. Ezzat , et al., “Deciphering the Therapeutic Potential of Trimetazidine in Rheumatoid Arthritis via Targeting Mi‐RNA128a, TLR4 Signaling Pathway, and Adenosine‐Induced FADD‐Microvesicular Shedding: In Vivo and In Silico Study,” Frontiers in Pharmacology 15 (2024): 1406939. 38919260 10.3389/fphar.2024.1406939 PMC11196411

[43] A. T. Eldmnawy , M. F. Tolba , H. Elghazaly , and S. A. Wahdan , “Trimetazidine Protects Against Doxorubicin‐Induced Chemobrain in Rats: Insights Into Energy Imbalance and Neuroinflammation,” European Journal of Pharmacology 1008 (2025): 178339. 41205975 10.1016/j.ejphar.2025.178339

[44] S. H. Mahmoud , S. M. H. Algenabi , A. Nather Seiwan , et al., “Trimetazidine Attenuates Ulcerative Colitis‐Linked Extrapyramidal Dysfunction by Mediated Dectin‐1/LRRK2/α‐Synuclein Autophagy Axis,” Microscopy and Microanalysis 32, no. 1 (2026): eozaf118. 10.1093/mam/ozaf118 41566671

[45] J. Frontiñán‐Rubio , E. Llanos‐González , S. García‐Carpintero , et al., “CoQ(10) Reduces Glioblastoma Growth and Infiltration Through Proteome Remodeling and Inhibition of Angiogenesis and Inflammation,” Cellular Oncology (Dordrecht) 46, no. 1 (2023): 65–77. 10.1007/s13402-022-00734-0 PMC9947058 36319818

[46] X. Hua , G. Hu , Q. Hu , et al., “Single‐Cell RNA Sequencing to Dissect the Immunological Network of Autoimmune Myocarditis,” Circulation 142, no. 4 (2020): 384–400. 32431172 10.1161/CIRCULATIONAHA.119.043545

[47] X. Hua , M. Bao , H. Mo , et al., “STING Regulates the Transformation of the Proinflammatory Macrophage Phenotype by HIF1A Into Autoimmune Myocarditis,” International Immunopharmacology 121 (2023): 110523. 37354779 10.1016/j.intimp.2023.110523

[48] D. Gu , M. Chen , Y. Yang , C. Lu , C. Li , and Y. Lin , “Targeting the PHD2/HIF‐1α/HO‐1 Pathway: A Key Role of Trimetazidine in Hypertensive Nephropathy,” Clinical and Experimental Hypertension 47, no. 1 (2025): 2563033. 41076558 10.1080/10641963.2025.2563033

[49] T. Peng , X. Tao , Z. Xia , et al., “Pathogen Hijacks Programmed Cell Death Signaling by Arginine ADPR‐Deacylization of Caspases,” Molecular Cell 82, no. 10 (2022): 1806–1820.e1808. 35338844 10.1016/j.molcel.2022.03.010

[50] W. T. Hsu , Y. H. Tseng , H. Y. Jui , C. C. Kuo , K. K. Wu , and C. M. Lee , “5‐Methoxytryptophan Attenuates Postinfarct Cardiac Injury by Controlling Oxidative Stress and Immune Activation,” Journal of Molecular and Cellular Cardiology 158 (2021): 101–114. 34087195 10.1016/j.yjmcc.2021.05.014

[51] Q. Long , L. Li , H. Yang , et al., “SGLT2 Inhibitor, Canagliflozin, Ameliorates Cardiac Inflammation in Experimental Autoimmune Myocarditis,” International Immunopharmacology 110 (2022): 109024. 35841866 10.1016/j.intimp.2022.109024

[52] Y. Zhang , Y. Xu , K. Zhou , G. Kao , M. Yan , and J. Xiao , “Hypoxia‐Inducible Transcription Factor‐1α Inhibition by Topotecan Protects Against Lipopolysaccharide‐Induced Inflammation and Apoptosis of Cardiomyocytes,” Biomedical Engineering Online 20, no. 1 (2021): 88. 34465337 10.1186/s12938-021-00923-2 PMC8407092

[53] M. Skrzypiec‐Spring , M. Kaczorowski , A. Rak‐Pasikowska , et al., “RhoA/ROCK Pathway Is Upregulated in Experimental Autoimmune Myocarditis and Is Inhibited by Simvastatin at the Stage of Myosin Light Chain Phosphorylation,” Biomedicine 12, no. 3 (2024): 596. 10.3390/biomedicines12030596 PMC10968043 38540212

[54] W. Gong , Y. Ma , A. Li , H. Shi , and S. Nie , “Trimetazidine Suppresses Oxidative Stress, Inhibits MMP‐2 and MMP‐9 Expression, and Prevents Cardiac Rupture in Mice With Myocardial Infarction,” Cardiovascular Therapeutics 36, no. 5 (2018): e12460. 30019466 10.1111/1755-5922.12460

[55] M. Sanoobar , S. Eghtesadi , A. Azimi , et al., “Coenzyme Q10 Supplementation Ameliorates Inflammatory Markers in Patients With Multiple Sclerosis: A Double Blind, Placebo, Controlled Randomized Clinical Trial,” Nutritional Neuroscience 18, no. 4 (2015): 169–176. 24621064 10.1179/1476830513Y.0000000106

[56] C. H. Lam , B. Zuo , H. H. Chan , et al., “Coenzyme Q10 Eyedrops Conjugated With Vitamin E TPGS Alleviate Neurodegeneration and Mitochondrial Dysfunction in the Diabetic Mouse Retina,” Frontiers in Cellular Neuroscience 18 (2024): 1404987. 38863499 10.3389/fncel.2024.1404987 PMC11165046

[57] B. A. Abdel‐Wahab , S. Y. Salem , H. M. Mohammed , N. A. Mohammed , and H. F. Hetta , “The Role of Vimentin, Connexin‐43 Proteins, and Oxidative Stress in the Protective Effect of Propranolol Against Clozapine‐Induced Myocarditis and Apoptosis in Rats,” European Journal of Pharmacology 890 (2021): 173645. 33098837 10.1016/j.ejphar.2020.173645

[58] S. S. Ramadan , F. A. El Zaiat , E. A. Habashy , et al., “Coenzyme Q10‐Loaded Albumin Nanoparticles Protect Against Redox Imbalance and Inflammatory, Apoptotic, and Histopathological Alterations in Mercuric Chloride‐Induced Hepatorenal Toxicity in Rats,” Biomedicine 11, no. 11 (2023): 3054. 10.3390/biomedicines11113054 PMC10669886 38002054

[59] D. A. Shabaan , N. Mostafa , M. M. El‐Desoky , and E. A. Arafat , “Coenzyme Q10 Protects Against Doxorubicin‐Induced Cardiomyopathy via Antioxidant and Anti‐Apoptotic Pathway,” Tissue Barriers 11, no. 1 (2023): 2019504. 34939895 10.1080/21688370.2021.2019504 PMC9870010

[60] K. Cai , H. Jiang , Y. Zou , et al., “Programmed Death of Cardiomyocytes in Cardiovascular Disease and New Therapeutic Approaches,” Pharmacological Research 206 (2024): 107281. 38942341 10.1016/j.phrs.2024.107281

[61] D. Mantle and B. A. Golomb , “Coenzyme Q10 and Xenobiotic Metabolism: An Overview,” International Journal of Molecular Sciences 26, no. 12 (2025): 5788. 40565253 10.3390/ijms26125788 PMC12193255

[62] H. Goel , N. Roma , M. Morgan , et al., “Trimetazidine in Cardiovascular Disease and Beyond: A Comprehensive Review,” American Journal of Cardiovascular Drugs 25, no. 4 (2025): 443–460. 40180780 10.1007/s40256-025-00724-1

[63] J. Wu , Y. Cui , W. Ding , J. Zhang , and L. Wang , “The Protective Effect of Macrostemonoside T From Allium Macrostemon Bunge Against Isoproterenol‐Induced Myocardial Injury via the PI3K/Akt/mTOR Signaling Pathway,” International Immunopharmacology 133 (2024): 112086. 38642441 10.1016/j.intimp.2024.112086

[64] M. L. Ndzie Noah , G. K. Adzika , R. Mprah , et al., “Estrogen Downregulates CD73/Adenosine Axis Hyperactivity via Adaptive Modulation PI3K/Akt Signaling to Prevent Myocarditis and Arrhythmias During Chronic Catecholamines Stress,” Cell Communication and Signaling: CCS 21, no. 1 (2023): 41. 36823590 10.1186/s12964-023-01052-0 PMC9948346

[65] H. M. Li , K. Y. Li , Y. Xing , et al., “Phenylephrine Attenuated Sepsis‐Induced Cardiac Inflammation and Mitochondrial Injury Through an Effect on the PI3K/Akt Signaling Pathway,” Journal of Cardiovascular Pharmacology 73, no. 3 (2019): 186–194. 30839512 10.1097/FJC.0000000000000651

[66] S. N. Rai , H. Dilnashin , H. Birla , et al., “The Role of PI3K/Akt and ERK in Neurodegenerative Disorders,” Neurotoxicity Research 35, no. 3 (2019): 775–795. 30707354 10.1007/s12640-019-0003-y

[67] Q. Yang , S. Li , Z. Zhou , et al., “Trimetazidine Mitigates High Glucose‐Induced Retinal Endothelial Dysfunction by Inhibiting PI3K/Akt/mTOR Pathway‐Mediated Autophagy,” Bioengineered 13, no. 3 (2022): 7515–7527. 35259050 10.1080/21655979.2022.2048993 PMC8974130

[68] F. Chu , K. Li , X. Li , L. Xu , J. Huang , and Z. Yang , “Graphene Oxide Ameliorates the Cognitive Impairment Through Inhibiting PI3K/Akt/mTOR Pathway to Induce Autophagy in AD Mouse Model,” Neurochemical Research 46, no. 2 (2021): 309–325. 33180247 10.1007/s11064-020-03167-z

[69] H. Kamranian , H. Asoudeh , R. K. Sharif , et al., “Neuroprotective Potential of Trimetazidine Against Tramadol‐Induced Neurotoxicity: Role of PI3K/Akt/mTOR Signaling Pathways,” Toxicology Mechanisms and Methods 33, no. 7 (2023): 607–623. 37051630 10.1080/15376516.2023.2202785

[70] S. A. Abuelezz and N. Hendawy , “Spotlight on Coenzyme Q10 in Scopolamine‐Induced Alzheimer's Disease: Oxidative Stress/PI3K/AKT/GSK 3ß/CREB/BDNF/TrKB,” Journal of Pharmacy and Pharmacology 75, no. 8 (2023): 1119–1129. 37315215 10.1093/jpp/rgad048

[71] K. Zhang , J. Gan , B. Wang , et al., “FGF21 Protects Against HFpEF by Improving Cardiac Mitochondrial Bioenergetics in Mice,” Nature Communications 16, no. 1 (2025): 1661. 10.1038/s41467-025-56885-9 PMC11829982 39955281

[72] S. Wen , K. Yang , Y. Bai , et al., “Investigating the Mechanism of Action of Schisandra Chinensis Combined With Coenzyme Q10 in the Treatment of Heart Failure Based on PI3K‐AKT Pathway,” Drug Design, Development and Therapy 17 (2023): 939–957. 37006723 10.2147/DDDT.S393995 PMC10065024

